# Cu_1–*x*_Fe*_x_*O: hopping transport and ferromagnetism

**DOI:** 10.1098/rsos.170339

**Published:** 2017-09-13

**Authors:** Mohd. Nasir, Rakibul Islam, Md. A Ahmed, Saniya Ayaz, Gautham Kumar, Sunil Kumar, C. L. Prajapat, Frederick Roussel, Sajal Biring, Somaditya Sen

**Affiliations:** 1 Department of Physics, Indian Institute of Technology Indore, Indore 453552, India; 2 Department of Metallurgy Engineering and Material Science, Indian Institute of Technology Indore, Indore 453552, India; 3 Unite Materiaux et Transformations (UMET)–CNRS UMR 8207, University of Lille-Sciences and Technologies, UFR de Physique, Bat P5, 59655 Villeneuve d'Ascq, France; 4 Department of Physics, University of Calcutta, Kolkata 700009, India; 5 Department of Electronic Engineering, Ming Chi University of Technology, New Taipei City, 8802, Taiwan, Republic of China; 6 Technical Physics Division, Bhabha Atomic Research Centre, Mumbai 400085, India

**Keywords:** electronic structure, X-ray photoelectron spectroscopy, valence state, hopping transport, room temperature ferromagnetism

## Abstract

Single phase, sol–gel prepared Cu_1*–x*_Fe*_x_*O (0 ≤ *x* ≤ 0.125) powders are characterized in terms of structural, electronic and magnetic properties. Using dielectric and magnetic studies we investigate the coupling of electron and spin. The electrical conductivities and activation energies are studied with increasing Fe content. Modelling of experimental conductivity data emphasizes a single hopping mechanism for all samples except *x* = 0.125, which have two activation energies. Hole doping is confirmed by confirming a majority Fe^3+^ substitution of Cu^2+^ in CuO from X-ray photoelectron spectroscopy studies (XPS). Such a substitution results in stabilized ferromagnetism. Fe substitution introduces variation in coercivity as an intrinsic magnetic property in Fe-doped CuO, and not as a secondary impurity phase.

## 1. Introduction

Monoclinic copper (II) oxide, CuO, is a p-type antiferromagnetic material with a band gap approximately 1.4 eV and Neel temperature approximately 230 K [1]. Considerable literature is available on transition metal (TM) doped/substituted CuO (e.g. Fe [2], Ni-doped CuO [3], Fe/Ni co-doped CuO [4] and Fe/Li co-doped CuO [5]). However, these reports are not in agreement with each other. Most probably this disagreement is due to compositional inhomogeneity in the compounds. Compositional inhomogeneity gives rise to inhomogeneous structure of the materials. Structural inhomogeneity, on the other hand, influences ionic separation. Inhomogeneity in ionic separation leads to different kinds of hybridization between dopant TM 3*d*, O 2*p* and Cu 3*d* electrons. This influences the double exchange interaction mechanism in a TM-ion doped CuO lattice [6]. Therefore, a careful structural analysis and electronic characterization is extremely important.

In Fe-doped CuO, Li *et al*. [7] studied room temperature ferromagnetism (RTFM). Dopant Fe^3+^-induced cation vacancies, ‘□’, in the lattice. The Fe–O–□ ferromagnetic coupling was stronger than superexchange between Fe–O–Cu. Park *et al*. [8] reported RTFM coupling among neighbouring Fe^3+^ ions mediated by carriers localized around oxygen vacancies. In Fe-doped CuO nanorods, Park *et al.* [8] and Manna & De [9] claimed RTFM due to shape anisotropy, mixed valency and non-Jahn–Teller properties of Fe ions. Wide controversies exist in explaining the observations, and the origin of RTFM is not yet clear. There is some experimental evidence of strong spin–phonon interaction in CuO [10,11], which needs to be taken into account.

We have synthesized nanocrystalline Cu_1*–x*_Fe*_x_*O (*x* = 0, 0.027, 0.055, 0.097 and 0.125) powders, with extreme care on homogeneity, by standard Pechini sol–gel process [12]. A sol–gel prepared sample allows proper mixing of the constituent ions. The structural goodness of these sol–gel synthesized samples has been elaborated with X-ray diffraction (XRD) and absorption studies. Homogeneity and phase purity has been ensured in these samples. In this report, we investigate the conduction mechanism and magnetic properties to understand RTFM in CuO by Fe doping.

## 2. Experimental methods

The XRD patterns of the nanoparticles were obtained using a Bruker D2 Phaser X-ray diffractometer with Cu-K_α_ (λ = 1.54 Å) source. SPECS high-resolution X-ray photoelectron spectroscopy (XPS) system with monochromatic Al-K_α_ X-ray (*hν* = 1.48 keV) primary radiation source (optimum energy resolution approx. 0.5 eV) was used to study the valence states. Broadband dielectric spectroscopy (Solarton Analytical—Ametek) was employed to investigate the electrical properties of Cu_1*–x*_Fe*_x_*O. Magnetic field-dependent magnetization was investigated using Quantum Design SQUID VSM (model SVSM-050).

## 3. Results and discussions

**Figure 1.**
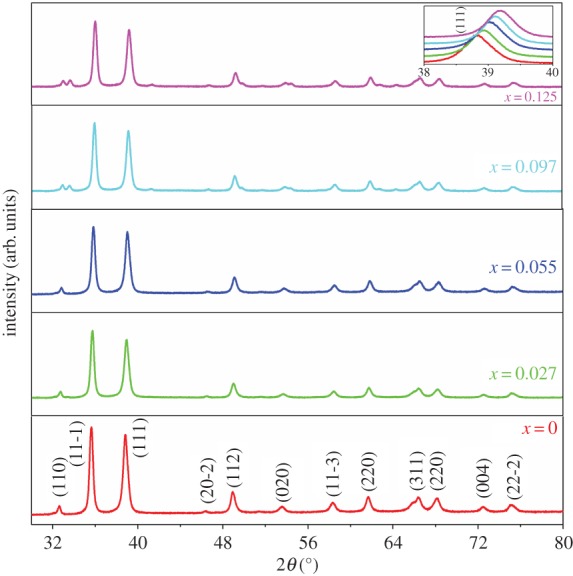
XRD pattern of Cu_1-*x*_Fe*_x_*O (0 < *x* < 0.125) samples revealing a systematic shift in (111) peak towards larger angles.

Structural studies using XRD reveal monoclinic single crystalline phase (figure 1). Shifting of (111) diffraction peak to higher angles (figure 1, inset) hints towards reducing lattice parameters. Reitveld refinement confirmed changes in lattice parameters. A continuous decrease in ‘a’ and ‘b’ was observed. On the other hand, ‘c’ showed a rapid decrease until *x* = 0.027 and thereafter became independent of substitution [13].

**Figure 2.**
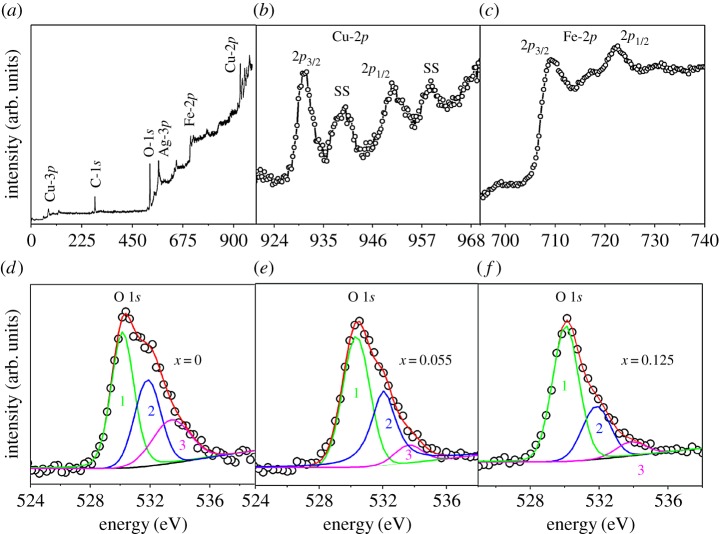
(*a*) XPS survey spectrum and high-resolution scans of (*b*) Cu 2*p* and (*c*) Fe 2*p* of Cu_0.875_Fe_0.125_O*.* The O 1*s* edge spectra of Cu_1-*x*_Fe*_x_*O samples; (*d*) *x* = 0, (*e*) *x* = 0.055 and (*f*) *x* = 0.0125.

X-ray photo electron spectroscopy (XPS) of the Cu_1-*x*_Fe*_x_*O (figure 2*a*) shows the presence of core-level lines of Cu 2*p*, O 1*s* and Fe 2*p*. The high-resolution Cu 2*p* doublet spectrum (figure 2*b*) shows Cu 2*p_3/2_* and Cu 2*p_1/2_* peaks at binding energies of 930.85 and 950.4 eV, respectively. Shake-up satellite (SS) features appear at approximately 939.73 eV and 958.7 eV. The satellite is a characteristic of Cu^2+^ valence states in copper halides having 3*d*^9^L (L for ligand) configuration [14]. This confirms Cu^2+^ valence states in these samples.

A Fe 2*p* doublet (Fe 2*p_1/2_* and Fe 2*p_3/2_*) is observed approximately at 722.7 eV and 710–714 eV, respectively (figure 2*c*). The 12.7 eV splitting in Fe 2*p* spectra is due to spin--orbit coupling. In the case of Fe 2*p_3/2_*, the binding energy is in the range 709.4–710.3 eV and 710.3–711.4 eV for Fe^2+^ and Fe^3+^, respectively [15,16]. The broadness of the Fe 2*p_3/2_* peak implies mixed valence states of majority Fe^3+^ and minority Fe^2+^. A shake-up satellite contribution is observed at approximately 717.59 eV. This is in close agreement with literature and indicates majority Fe^3+^ valence state [17]. The absence of peak at 706–707 eV rules out the presence of Fe metallic clusters. Hence, magnetism should not be attributed to interstitial or externally existing iron clusters.

The O 1*s* spectra (figure 2*d*–*f*) of Cu_1-*x*_Fe*_x_*O samples are asymmetric. The asymmetric O 1*s* peak has been discussed in the literature to arise from three contributions: peaks appearing at approximately 533.83 eV (peak_3_: related to H_2_O adsorbed on the surface of the sample), approximately 531.89 eV (peak_2_: caused by either OH^−^ hydroxyl groups or by chemisorbed molecular oxygen ${\text{O}}_{2}^{-}$) and approximately 530.15 eV (peak_1_, associated with oxygen O^2−^ in CuO lattice [5,18,19]). We have fitted O 1*s* edge with three Gaussian peaks and found that the relative area under peak_1_ is increasing with respect to peak_2_ and peak_3_ with increasing x. Hence, the oxygen contained in the lattice is increasing compared with the other two contributions. Note that EXAFS (extended X-ray absorption fine structure) analysis of these materials also reveals reduction in oxygen deficiency with increased Fe substitution [13]. The reducing coordination numbers of metal ions clearly indicate the same.

**Figure 3.**
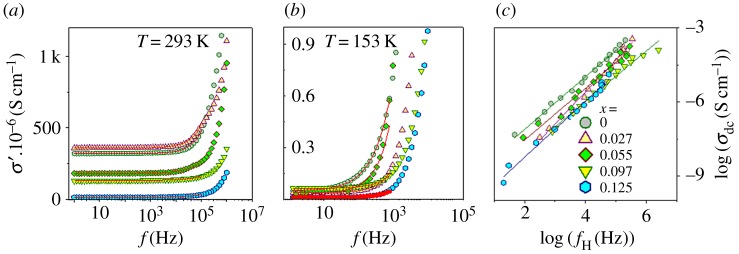
Frequency spectra of the real conductivity (*σ′*) at (*a*) 293 K, (*b*) 153 K and (*c*) Barton--Nakajima--Namikawa (BNN) plots for Cu_1*–x*_Fe*_x_*O (0 ≤ *x* ≤ 0.125). The solid lines are obtained from the Jonscher Law fit of complex conductivities.

**Figure 4.**
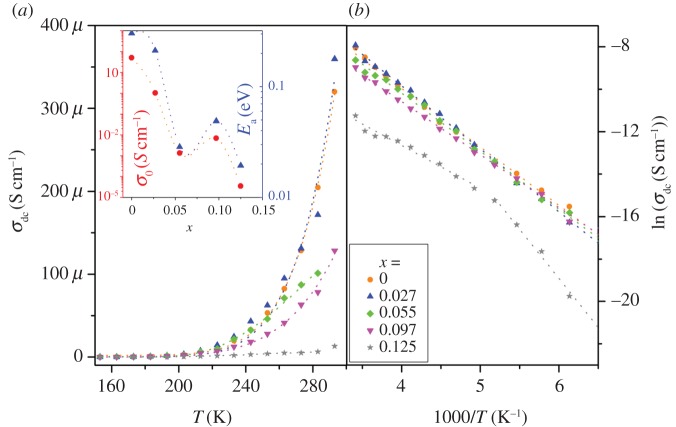
Temperature dependence of (*a*) DC conductivity, *σ*_dc_ versus *T* and (*b*) log(*σ*_dc_) versus 1000/*T* for Cu_1*–x*_Fe*_x_*O (0 ≤ *x* ≤ 0.125). The dotted lines represent fitted spectra.

**Figure 5.**
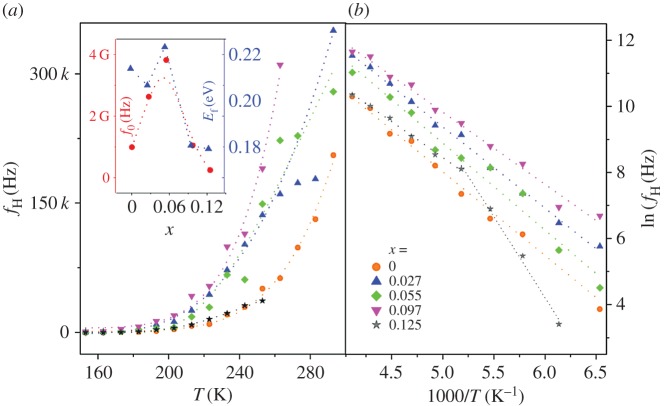
Temperature dependence of (*a*) hopping frequency, *f*_H_ versus *T* and (*b*) log(*f*_H_) versus 1000/*T* for Cu_1*–x*_Fe*_x_*O (0 ≤ *x* ≤ 0.125). The dotted lines represent fitted spectra.

The frequency-dependent real part *σ*′ of the complex electrical conductivity σ*( *f*) is connected to the imaginary part *ε′′* of complex permittivity ε*( *f*) as [20] *σ*′ = 2*πfε*_0_*ε′′*, where, *f* denotes the experimental frequency of the harmonic voltage applied to the specimen. The real part of frequency dependence of electrical AC conductivity (*σ*′( *f*)) for all compositions at temperatures 153 K and 293 K has been plotted (figure 3*a*,*b*). AC conductivity, *σ*′(ƒ) increases with increasing frequency above a characteristic onset frequency *f*_H_. Below *f*_H_ it is non-variant with dispersion at higher frequencies. The non-variant region at very low frequencies can be compared with the DC conductivity *σ*_dc_. There is no significant change in conductivity in a wide frequency range. The dispersion shifts to lower frequencies with decreasing temperatures. With increasing Fe content, and decreasing temperature *σ*′( *f*) decreases. Hopping carriers interact with inherent defects or disorderedness in the material especially in low frequency regime. Jonscher's universal dielectric response (UDR) model [21], originates from such interactions, given by:

3.1 $$\sigma \prime (\;f)=\sigma_{\mathrm{dc}}\left[1+{\left(\frac{f}{f_{H}}\right)}^{n}\right],$$

where *σ*_dc_ is the DC conductivity, *f*_H_ is onset frequency of the hopping process and *n* is a frequency exponent parameter in the range 0 ≤ *n* ≤ 1. The frequency-dependent real part of the electrical conductivities of the Cu_1-*x*_Fe*_x_*O samples have been modelled (figure 3*a*,*b*) using Jonscher's UDR model. The temperature dependence of *σ*_dc_ and *f*_H_ was extracted from the above model and has been plotted with temperature (figures 4 and 5). With increasing temperature, *σ*_dc_ increases nonlinearly, revealing semiconducting nature of the samples. Hopping conduction occurs through the neighbouring sites in the nearest-neighbour-hopping (NNH) conduction model. The activation energy can be analysed by the Arrhenius equation [22],

3.2 $$\sigma_{\mathrm{dc}}(T)=\sigma_{0}\;\exp\left(\frac{-E_{a}}{K_{B}T}\right),$$

where *σ*_0_ is a constant representing the DC conductivity at *T *→ ∞, *K*_B_ is the Boltzmann constant and *E*_a_ is the activation energy for hopping conduction. *σ*_dc_ was fitted with NNH model. Arrhenius model can be better understood by plotting log(*σ*_dc_) (S cm^−1^) against 1000/*T* (K^−1^) (figure 4*b*). A linear nature of this plot ensures Arrhenius behaviour. It was observed that for lower substitution the plots were extremely linear. However, for *x* ≥ 0.055, some nonlinearity was observed. For *x* = 0.125, the plot was extremely nonlinear. The non-Arrhenius behaviour is rectified in the literature by Vogel–Tamman–Fulcher (VTF) model, given by

3.3 $$\sigma_{\mathrm{dc}}(T)=\sigma_{0}\;\exp\left(\frac{-E_{a}}{K_{B}(T-T_{0})}\right),$$

where *T*_0_ is the Vogel temperature. In VTF model, the migration of ions depend on such defects which arise from structural modification within the system [23].

The VTF equation provides a good fit to the DC conductivity for all samples. For low *x*, *T*_o_ → 0, but for *x* > 0.055, *T*_o_ is approximately 150 K. Thus both Arrhenius and VTF models can explain samples with *x* ≤ 0.27, but beyond *x* ≥ 0.055 the samples are better explained by VTF model. This transformation is most probably due to ionic motion in which Fe plays an important role facilitated by decreasing grain size of the crystallites. Fe^3+^ ion having a different ionic radius is a most likely source of internal strain. It not only creates localized defects but also reduces single crystal domain size within the same Cu_1-*x*_Fe*_x_*O grain as already reported in SEM studies [12]. *E*_a_ and *σ*_0_ decreases with increasing substitution (figure 4*a*, inset). The decreasing trend of σ_dc_ of these samples was also directly calculated from *I*–*V* characteristics and was reported previously [12].

CuO is a p-type material. Fe^3+^ ions provide excess electrons to the lattice. However, due to significantly p-type CuO host these excess electrons cannot improve the conductivity. It was reported from Hall measurements that p-type carrier concentration decreased [12], reducing the net conductivity of the material with increasing substitution. Reduction in oxygen vacancies with increasing Fe content was found from EXAFS analysis [13] due to extra charge of Fe^3+^ than Cu^2+^ ions. The reducing domain sizes generate more domain walls, and lattice becomes more defected with increasing substitution. Thus with increasing substitution mobility is probably reduced. Thus, the reduction in *σ*_0_ and *E*_a_ is most probably due to a combination of reduced carrier concentration as well as mobility.

Both Arrhenius and VTF models are single-activation energy models. Good fits to the experimental data using these models emphasize a single conduction mechanism present in all the substituted samples. However, only for *x* = 0.125, from the ln *σ*_dc_ versus 1000/*T* plots it seems that a double activation model may also fit the data with activation energies approximately 0.2 and 0.4 eV.

Similar to *σ*_dc_, the hopping frequency *f*_H_(*T*) data were fitted to the Arrhenius model (figure 5*a*,*b*): *f*_H_(*T*) = *f*_0_ exp(−*E*_H_/*K*_B_*T*), where *f*_0_ is a constant and *E*_H_ is the activation energy of hopping frequency of carriers. The data were also fitted to the VTF model, defined as, *f*_H_(*T*) = *f*_1_exp[−*E*_H_/*k*_B_(*T* − *T*_0_)], where *f*_1_ and *T*_0_ are fitting parameters. Similar to the σ_dc_ data it is noted that *f*_H_ follow the same trend as a function of temperature. At very low substitution the Arrhenius nature prevails but as substitution increases a VTF model dominates. *E*_H_ and *f*_0_ decreases with increased Fe substitution (figure 5*a*, inset).

To explain the relationship between *σ*_dc_ and *f*_H_, log(*σ*_dc_ (S cm^−1^)) versus log(*f*_H_ (Hz)) is plotted, which is shown in figure 3*c*. The linear behaviour of the plots follows the Barton–Nakajima–Namikawa (BNN) relation. It is noteworthy that upon Fe substitution the conductivity decreases instead of increasing, once again emphasizing the point that proper substitution has happened in the samples.

**Figure 6.**
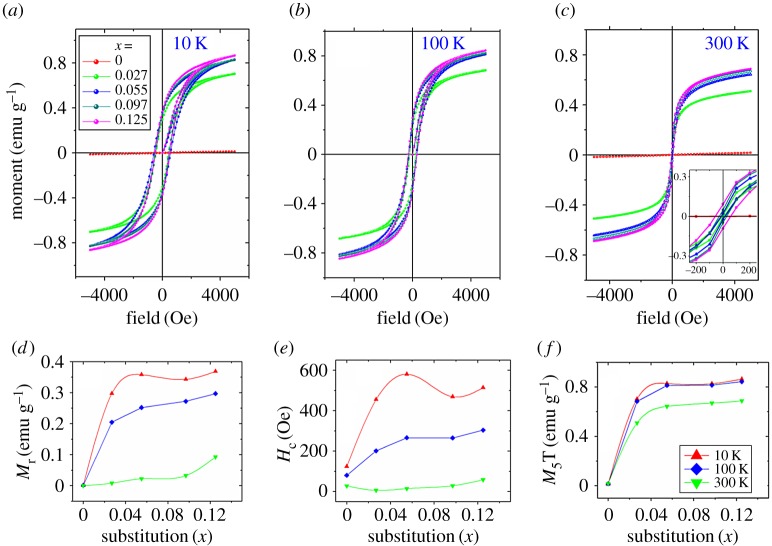
Ferromagnetic hysteresis loops (M–H) at (*a*) 10 K, (*b*) 100 K, (*c*) 300 K, (*d*) remnant magnetization (*M*_r_), (*e*) coercivity (*H*_c_) and (*f*) magnetization (M_5_T) versus substitution at 5 T field for Cu_1*–x*_Fe*_x_*O (0 ≤ *x* ≤ 0.125) samples.

The field-dependent magnetization [M versus H] curves for Cu_1-*x*_Fe*_x_*O samples (figure 6*a*–*c*) at different temperatures are ferromagnetic in nature. Saturation magnetization, *M*_S_, is not achieved up to 5000 Oe. The remnant magnetization, *M*_r_, and coercive field, *H*_c_, increase linearly with substitution (figure 6*d*,*e*) indicating stronger ferromagnetic exchange interaction. The varying nature of *H*_c_ and *M*_r_ along with M_5T_ (at 5 T field) with substitution is a proof of magnetization being not due to impurity phase of iron oxides or metallic clusters of Fe but due to proper substitution.

Oxidation states of Fe ions in CuO play a critical role in magnetic properties of CuO. It has been mentioned that in the Fe^3+^ substituted samples, the p-type nature is retarded while the oxygen deficiency is reduced. This type of change is not expected in the case of Fe^2+^ substitution. In the case of Fe^2+^ oxygen vacancies could have increased as Fe^2+^ has the same charge and has a crystal radius approximately 0.77 Å, which is about 8.5% more than Cu^2+^ ion. As a result oxygen vacancy mediated ferromagnetism could have been possible. The exchange interaction would then have been through an electron trapped at the oxygen vacancy site, *V*_o_ [24]. The M1–*V*_o_–M2 interaction [25] is generally ferromagnetic in the case of Fe^3+^–*V*_o_–Fe^3+^ [24]. In the case of Fe^2+^–*V*_o_–Fe^2+^, it is weaker but still ferromagnetic. However, in a homogeneous solid solution to find neighbouring Fe^2+^ or Fe^3+^ ions is less probable. Moreover, we have seen predominant increase of oxygen content with substitution. Hence, be it Fe^3+^ or Fe^2+^, an enhancement of ferromagnetism, mediated by oxygen deficiencies, seems inappropriate. Note that in pure CuO, very weak ferromagnetism is observed due to Cu^2+^–*V*_o_–Cu^2+^ interactions. However, Cu–O–Cu weak antiferromagnetic ordering dominates. This in fact is present in all samples, thereby not letting the hysteresis curves saturate. Hence we have plotted the composition dependence of M_5T_, the magnetic moment at 5 T, after the loop closure.

Mediated through Fe^3+^–O^2−^–□ [7], ferromagnetism is enhanced, where □ is a cation vacancy. A cation vacancy is associated with anion vacancy in the lattice during synthesis. In a pure undoped material the ratio of the two should be equal to maintain charge neutrality. Initially, the oxygen vacancies will be reduced by Fe^3+^ incorporation. Further incorporation invites excess oxygen in the lattice, thereby generating cation vacancies. Hence the increased magnetism may be due to such cation deficiencies.

Metal–oxygen–metal double exchange interactions have played an important role in magnetism. An increase in oxygen content thereby makes such exchange integrals stronger. Fe^3+^ substitution opens up a better chance of Fe^3+^–O^2−^–Cu^2+^ superexchange or a Fe^3+^–O^2−^–Cu^2+^–O^2−^–Fe^3+^ double superexchange phenomena. Note that the replacement of Cu^2+^ by Fe^3+^ ion in CuO enhances the magnetic moment. The spin of Fe^3+^ (5/2) being more than that of Cu^2+^ (1/2) ion, may be the reason. It has been demonstrated that in Fe_2-x_Cu_x_O_3_ ferromagnetism is enhanced with increasing Cu content [26]. In *x* = 1, i.e. FeCuO_3_ sample, maximum ferromagnetism has been observed. This enhancement hints at a stronger ferromagnetism in Cu–O–Fe than Fe–O–Fe couplings. Thereby in Fe-substituted CuO such strong interactions may also contribute to the enhanced ferromagnetism.

The influence of TM doping on magnetic properties of CuO was also studied by Wesselinowa [27], and it was found that the exchange interaction *J_ij_* = *J*(*r_i_* − *r_j_*) depends on the distance between the spins. The smaller the lattice parameters, the shorter are the interionic distances and thereby stronger is the exchange interaction. The crystal radius of Fe^3+^ (0.63 Å) is lesser than Cu^2+^(0.71 Å). Hence substituted lattice tends to contract. XRD results revealed that lattice parameters have reduced with increasing Fe substitution. As calculated by Wesselinowa, the Fe–O–Cu superexchange increases thereby increasing the ferromagnetism in the material. In these samples, we observe proper experimental evidence of the same, and link the results to the role of increasing oxygen and Fe^3+^ content. Lattice parameters are expected to decrease with reducing temperature. Thus the interatomic separations should decrease. This may be the reason behind increasing ferromagnetism with decreasing temperature.

The spin–phonon coupling makes an important contribution to exchange integral. Wesselinowa [27] reported the Neel temperature increases due to the larger exchange interaction in CuO. But this interaction was weaker than the case where spin–phonon coupling was added to the Hamiltonian. The spin–phonon interaction renormalizes and enhances the exchange interaction. Chen *et al*. [10] and Kuz'menko *et al*. [11] also report the same. In the case of Fe–O–Cu ferromagnetic interaction this may also contribute considerably thereby increasing the Curie temperature. Phonon modes are dependent on lattice structure. Hence, with modifications in the lattice parameters, spin–phonon interaction is also dependent on such changes.

Note that the remnant magnetization and coercive fields increase with increasing substitution and decreasing temperature. With increasing substitution the long-range coupling between Fe–O–Cu and the Fe–O–□ increases. Also, with reducing temperature interatomic spacing decreases and may be responsible for enhanced ferromagnetism due to spin–phonon coupling as well as Cu–O–Fe exchange interactions.

## 4. Conclusion

Monoclinic single phase Fe^3+^ substituted Cu_1*–x*_Fe*_x_*O (*x* = 0, 0.027, 0.055, 0.097, 0.125) have been examined by XPS, electrical and magnetic studies. Impurity phases of Fe metallic cluster and Fe_2_O_3_ has been ruled out from XPS, structural studies, electrical conductivity and magnetic measurements. The electrical conductivities and activation energies are found to decrease with increase in Fe content. The experimental data can be modelled using a single hopping mechanism for all samples except *x* = 0.125, which has two activation energies. Weak ferromagnetic behaviour has been observed at room temperature for all the samples. Magnetism increases with decreasing temperature and increased Fe substitution. The increasing remnant magnetization and coercive fields in substituted CuO results from increasing amount of Fe–O–Cu and Fe–O–□ interactions, where, □ are cation vacancies created due to excess Fe^3+^ substitution. These exchange interactions are sometimes stronger that a normal Fe^3+^–O^2−^–Cu^2+^ superexchange or a Fe^3+^–O^2−^–Cu^2+^–O^2−^–Fe^3+^ double superexchange interaction, thereby enhancing the ferromagnetism. This study motivates a future size-dependent magnetic study of Fe-doped CuO.

## Data accessibility

Our data are deposited at Dryad: (http://dx.doi.org/10.5061/dryad.vv121) [28].
